# Promising the potential of β-caryophyllene on mercury chloride–induced alteration in cerebellum and spinal cord of young Wistar albino rats

**DOI:** 10.1007/s00210-024-03268-4

**Published:** 2024-07-12

**Authors:** Ahmad Yahyazadeh, Fatih Mehmet Gur

**Affiliations:** 1 Department of Histology and Embryology, Faculty of Medicine, Karabuk University, Karabuk, Turkey; 2 Department of Histology and Embryology, Faculty of Medicine, Nigde Ömer Halisdemir University, Nigde, Turkey

**Keywords:** Cerebellum, β-Caryophyllene, Immunohistochemistry, Mercury chloride, Rat, Spinal cord

## Abstract

Mercury chloride (ME) is a chemical pollutant commonly found in the environment, which can contribute to undesirable health consequence worldwide. The current study investigated the detrimental impact of ME on the cerebellum and spinal cord tissues in 6–8-week-old female rats. We also evaluated the neuroprotective efficacy of β-caryophyllene (BC) against spinal and cerebellar changes caused by ME. Thirty-five young Wistar albino rats were randomly chosen and assigned into five groups: control (CO), olive oil (OI), ME, BC, ME + BC. All samples were analysed by means of unbiased stereological, biochemical, immunohistochemical, and histopathological methods. Our biochemical findings showed that SOD level was significantly increased in the ME group compared to the CO group (*p* < 0.05). We additionally detected a statistically significant decrease in the number of cerebellar Purkinje cells and granular cells, as well as spinal motor neuron in the ME group compared to the CO group (*p* < 0.05). In the ME + BC group, the number of Purkinje cells, granular cells, and spinal motor neurons was significantly higher compared to the ME group (*p* < 0.05). Decreased SOD activity in the ME + BC group was also detected than the ME group (*p* < 0.05). Immunohistochemical (the tumour necrosis factor-alpha (TNF-α)) and histopathological examinations also exhibited crucial information in each of the group. Taken together, ME exposure was associated with neurotoxicity in the cerebellum and spinal cord tissues. BC treatment also mitigated ME-induced neurological alteration, which may imply its potential therapeutic benefits.

## Introduction

Mercury is a heavy metal that can be found in water, air, and soil and is toxic to all living organisms. In nature, mercury can exist in a variety of forms: elemental (e.g. mercury vapour), inorganic (e.g. mercury chloride, ME), and organic (e.g. methyl mercury) (Kim et al. 2016; Yahyazedeh et al. 2017). Of these, inorganic mercury is the most prevalent. High potential for mercury exposure may result from amalgam, some vaccines, lotions, and skin medications containing mercury, consumption of seafood, fluorescent lamps, thermometer factories, and gold mining (Do et al. 2017; Yahyazedeh et al. 2017; Teixeira et al. 2018; Owoeye et al. 2019; Shalan 2022). After exposure, mercury can accumulate in several vital organs such as the intestine, brain, liver, placenta, and kidney (Bridges and Zalups 2017). As one of the most common forms in combination with other elements, inorganic mercury contributes substantially to environmental pollution and thus has harmful consequences at various concentrations. The gastrointestinal tract absorbs dietary inorganic mercury, which is subsequently transported to internal organs (Patrick 2002; Owoeye et al. 2019). The degree of tissue damage or biological dysfunction depends on the dose, time, and manner of delivery (Denny and Atchison 1996). Mercury’s biological and toxicological effects are also contingent upon its form.

Of the inorganic mercury forms, ME is thought to be a toxic chemical compound. After being metabolized in the liver, ME can pose serious risks to the brain tissues (Zulaikhah et al. 2020). Potential toxicity of ME is attributed to its high water solubility, ease of absorption, and easy binding to proteins (Lorscheider et al. 1995; Zulaikhah et al. 2020). Its toxicity is additionally linked to its strong affinity for the SH groups found in biomolecules such as sulfhydryl proteins and glutathione (Hansen et al. 2006). After binding to biomolecules, ME circulates in lymph or serum and then transmits to the central nervous system (CNS) (Lorscheider et al. 1995). Adverse biochemical and clinical effects associated with exposure to ME can also cause major problems (Abd Elghani et al. 2020). Furthermore, by upsetting the calcium equilibrium, contamination with mercury leads to disorders in a biological system (Sharma et al. 2005). Previous studies have connected the administration of ME to an increased risk of neurodevelopmental and sensory difficulties, liver alteration, kidney failure, visual impairment, coagulation abnormalities, and muscle weakness (Fernandes Azevedo et al. 2012; Bjorklund et al. 2018; Hazelhoff et al. 2018; Yang et al. 2020). This substance with low liposoluble property has been found to negatively impact neuronal homeostasis in the CNS (Clarkson and Magos 2006). An earlier investigation documented that the deposition of ME in the brain tissues led to impairment in motor function (Teixeira et al. 2014). Additionally, ME’s interaction with CNS may result in irreversible disruption to the brain’s fundamental functioning and cognitive impairment, as well as central auditory system problems (Huang et al. 2008). As a conduit between the brain and other parts of the body, the spinal cord tissues exposed to ME may undergo structural and functional alterations (Correa et al. 2020). Consequently, impairment of the spinal cord may result in a detrimental impact on the brain.

Reactive oxygen species (ROS) production can rise when oxidative balance is compromised, which can then result in oxidative damage. One reason for this is the antioxidant defence system’s impairment after ME treatment (Ahmad and Mahmood 2019). As a crucial modulator of inflammatory reactions, proinflammatory cytokines have also been implicated in the pathogenesis of several biological disorders (Hashemizadeh et al. 2022). According to clinical studies, oxidative stress, inflammation, and apoptosis have been associated with the biological implications of ME (Zhang et al. 2017). Experimental studies have documented the evidence of oxidative damage to the brain and spinal cord after ME exposure (Ferraro et al. 2009; Correa et al. 2020). Moreover, scientific evidence has been shown for an association between prolonged exposure to ME and serious degradation of essential macromolecules, specifically DNA (Mojica-Vazquez et al. 2019). Exogenous antioxidant delivery has been reported to be the potential to alleviate ME-induced complication by fortifying the body’s antioxidant defences (Al-Taee et al. 2019).

A class of phytochemicals known as phytocannabinoids has received great attention recently. β-Caryophyllene (BC) (C15H24), a phytocannabinoid, mostly exists as trans-caryophyllene with trace amounts of its oxidation derivative and isomers (Ullah et al. 2021). Spices are rich in BC, notable for its nutritional availability and safety. BC’s potential therapeutic advantages have been suggested to stem from its anticancer, apoptotic, anti-inflammatory, antioxidant, antispasmodic, and anticonvulsant capabilities (Leonhardt et al. 2010; Baldissera et al. 2017; Al-Taee et al. 2019; Refaat and El-Boshy 2022). Previous research studies have revealed that the administration of BC can mitigate unfavourable consequences arising from ME in the biosystem (Kanojia et al. 2021).

It is impossible to completely avoid environmental exposure to potentially hazardous compounds. Thus, an efficient approach is needed to prevent and/or minimize their health-related threats. Furthermore, there is no comprehensive research that addresses reversing the CNS alterations caused by environmental pollutants. Hence, this research proposed to investigate the detrimental impact of ME on the spinal cord and cerebellum tissues in female Wistar albino rats. Another aim was to survey the BC’s efficacy in ameliorating the alteration induced by ME. To achieve the accurate data that corroborate each other, we employed immunohistochemical, stereological, biochemical, and histopathological techniques.

## Materials and methods

### Chemical and reagents

#### Animals and experimental protocol

Thirty-five Wistar albino rats, 6–8 weeks old and weighing 130–150 g, were utilized in this investigation. The Karabuk University Laboratory Animal Ethics Committee awarded ethical approval for the current research (NO: 2022/09/16). All rats were housed in plastic cage under 12-h day/night with 21–24 °C and 45–55% humidity. During the experiment, rats were also given unlimited access to a standard chow diet and tap water. Our study’s in vivo stages and subject monitoring were completed at the Karabuk University Experimental Animal Care and Research Unit. Prior to the conduct of the study, subjects were randomly assigned into five groups of seven rats as follows:

1. Control (CO): Rats were fed distilled water orally for the duration of the 14-day trial.
2. Olive oil (OI) group: Rats were fed OI orally for 14 days.
3. Mercury chloride (ME): Rats were administered orally 4 mg/kg ME (a purity ≥ 99.5%, ISOLAB chemicals, Cat. No: 947.026.0250) dissolved in olive oil for 14 days (Owoeye et al. 2019).
4. β-Caryophyllene (BC) group: Rats were treated orally with 200 mg/kg BC (a purity of ≥ 80%, Sigma-Aldrich Cat. No: W225207) dissolved in distilled water for 14 days (Refaat and El-Boshy 2022).
5. Mercury chloride + β-caryophyllene (ME + BC) group: Rats were treated orally with 4 mg/kg ME for 14 days. An oral dose of 200 mg/kg BC was additionally administered to each animal during the 14-day experiment.

At the end of the experiment, all subjects were anaesthetized intraperitoneally with 80 mg/kg ketamine and 10 mg/kg xylazine. Blood samples were taken intracardially and then transferred into heparinized tubes. After euthanizing animals using exsanguination, all the bones surrounding the brain and spinal cords were removed. The cervical spinal cord and cerebellar tissues were then promptly extracted for immunohistochemical, stereological, and histopathological examination. All tubes were also centrifugated at 3000 rpm for 20 min, and their serums were collected and frozen at − 80 °C for subsequent biochemical analysis.

### Histological procedure

Fixation of dissected samples, the cervical spinal cords and cerebella, was carried out by immersing them in a 10% neutral formalin solution at + 4 °C for 24 h to preserve the tissue’s architectures (Weli and Yahyazadeh 2023; Yahyazadeh et al. 2024). Samples were then washed out in running water to remove excessive fixative. Subsequently, tissue processing was performed to provide sufficient rigidity for the cutting of thin sections. For this purpose, the samples were first dehydrated by passing samples through a graded alcohol series. Xylene was subsequently employed to clear the tissues via removing the alcohol. Thereafter, impregnation was carried out by embedding the samples in paraffin, a suitable medium (Balcioglu et al. 2021; Gur et al. 2021). Paraffin-embedded tissues were cut into 5-µm-thick sections using a rotary microtome for histological and immunohistochemical examination (Galileo SDSGA9000, Italy). These sections were sampled based on the systematic and random technique. Before mounting the histological sections on a slide, the wrinkles of them vanished by being placed into the water bath at 40 °C. Before being stained, excess paraffin was removed from the slides by incubating them in an oven at 60 °C overnight. The slides of the spinal cord and cerebellar tissues then underwent rehydration using xylene and graded alcohol series, as well as tap and distilled water. Subsequently, anti-TNF-α antibody and haematoxylin and eosin dye were used for immunohistochemical and histological staining of all sections. Finally, the sections were photographed using an Olympus BX-53 light microscope (Tokyo, Japan) and a digital camera (DP 80, Olympus).

### Analysis of biochemistry

Superoxide dismutase (SOD) assay kit (Relassay, Turkey) was used to determine serum SOD level in accordance with the manufacturer’s instruction. SOD, a major antioxidant enzyme and indicator for oxidative stress, can protect cells against oxidative damage to the variety of biomolecules via scavenging free radicals (Zheng et al. 2023). In other words, detoxification of ROS is the main biological function of SOD serving as a first line of defence system within cells. In fact, SOD activity in the body can be impacted by the oxidative stress induced by environmental contaminants; therefore, the measurement of SOD level was taken into consideration. This kit uses a tetrazolium salt for the detection of superoxide radicals generated by xanthine oxidase and hypoxanthine. Briefly, SOD standard and serum specimens were pipetted into the wells. After adding 20 µl of xanthine oxidase, the well plate was incubated for 30 min at room temperature to observe the colour change of the samples. Finally, spectrophotometric analysis was carried out using a UV–Vis spectrophotometer (Shimadzu UV-MINI 1240; Shimadzu; Istanbul, Turkey) at 440 nm.

### Stereological study

**Fig. 1:**
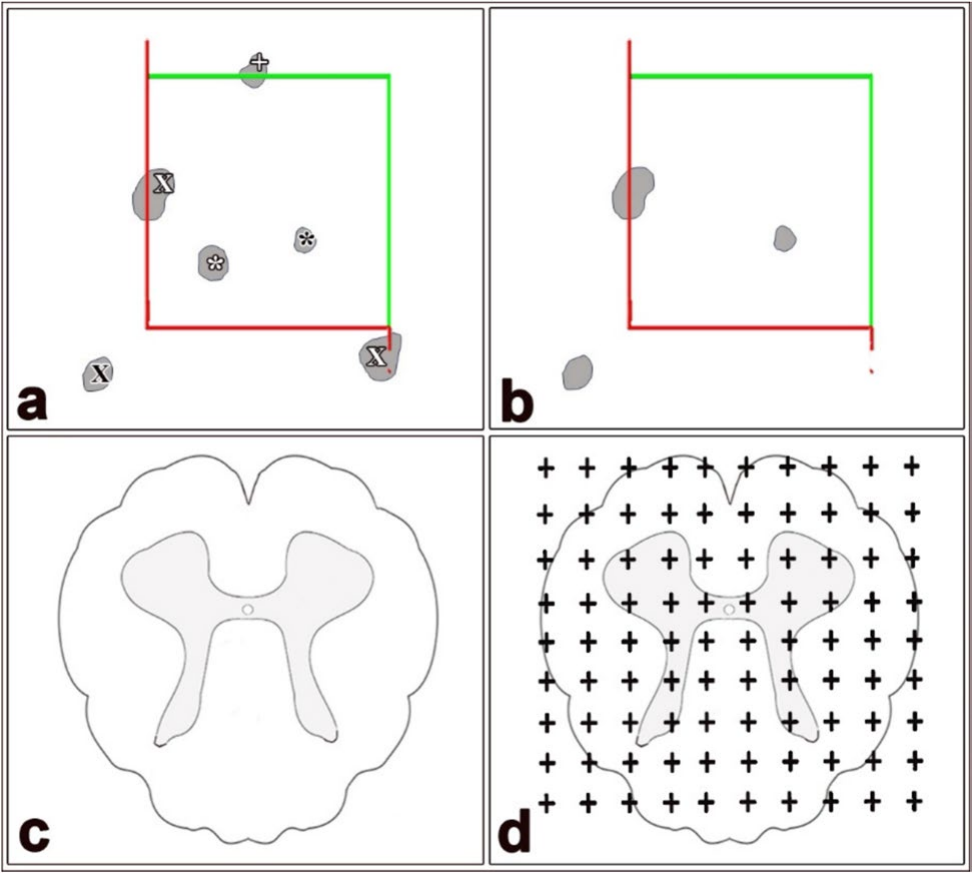
The stereological procedures of and the physical disector (**a** and **b**); the reference section (**a**); the look-up section (**b**); the Cavalieri principle (**a** and **b**); white X, particles touching the red exclusion lines; black asterisk, particle within the counting frames in both reference and look-up sections; white asterisk, particle within the counting frames in the reference section; + , particle touching the green inclusion line in the reference section; black X, particles located outside the counting frame in both reference and look-up sections

The physical disector technique was preferred to estimate the number of neurons in the cerebellar and cervical spinal cord tissues. The Cavalieri method was also employed to calculate the mean volumes of the regions of interest in the spinal cord and cerebella (Fig. 1). To estimate the number of neurons, we first calculated the mean volume of the cervical spinal cord and cerebellar tissues.

The Cavalieri method was used to calculate the mean volumes of cerebellar and spinal cord white matter, grey matter, spinal cord, and cerebellum, as well as volume fraction ratios of all parameters (Fig. 1c, d). To apply this method, the point-counting grid with appropriate space of points was employed. Designing the density of the grid point was carried out using a pilot study. Briefly, the point-counting grid was placed randomly on microphotographs taken from the cervical spinal cord and cerebellar sections. The number of points hitting the region of interest was counted and then multiplied by section thickness. The area of all parameters in the spinal cord and cerebellar tissues was calculated as follows (Aktas and Yahyazadeh 2022):

$$\text{Area}(A)=a\left(p\right)\times \sum P$$

where ‘‘*A*’’ is the area related to the white matter, grey matter, spinal cord, and cerebellar tissues, ‘‘Σ*P*’’ is the number of points, and ‘‘*a*(*p*)’’ is the area of point interval. The mean volumes of all parameters were estimated as follows:

$$\Sigma V=\Sigma A\times t$$

where ‘‘Σ*V*’’ is the mean volume of the spinal cord or cerebellar tissues, ‘‘Σ*A*’’ is the area of the regions of interest, and ‘‘*t*’’ is the total thickness of section.

The number of Purkinje cells, granular cells, and motor neurons was calculated using the physical disector technique, which is one of the unbiased stereological methods conducted on the consecutive sections (Fig. 1c, d) (Yahyazadeh et al. 2020). Briefly, the disector pairs were obtained from the spinal cord and cerebellar tissue using a systematic random sampling method. A pair of sections was defined as the reference section and the other as the look-up section. The unbiased counting frames were superimposed on the pairs of parallel and adjacent sections, then neurons were evaluated according to the disector counting rules. Neurons that were observed in the reference section but not in the look-up section were counted. Moreover, particles that do not touch the forbidden edges of the counting frame were accepted for counting. The numerical density of neurons (Purkinje cells, granular cells, motor neurons) was estimated using the following formula (Yahyazadeh et al. 2020):

$$N\text{v}=\frac{\sum Q-}{\sum V\text{ disector}}$$

where “Σ*Q*” is the number of neurons counted in the disector volume, and “Σ*V* disector” is the total volume of the disector frames in the reference sections. Finally, the number of neurons was calculated as follows:

$$N \left(\text{Number of neurons}\right)={N}_{\text{V}}\times {V}_{\text{ref}}$$

where “*V*_ref_” is the mean volume of the spinal cord and cerebellar tissues, and *N*_v_ is the numerical density of neurons.

### Immunohistochemical analysis

The immunostaining of samples was performed using anti-TNF-α antibody (ab220210; Abcam, Cambridge, MA), a key inflammatory marker. Antigen retrieval technique was applied to tissue sections with 4-µm thickness after deparaffinization (Gur and Timurkaan 2016a). To block endogenous peroxidase in the tissues, the tissues were incubated with 3% hydrogen peroxide in methanol for 5 min and subsequently underwent the procedures specified in our previous publications (Aktas and Gur 2022; Gur 2022). Tissue sections were then incubated overnight in a humid chamber at + 4 °C with a primary antibody diluted 1/200 in PBS to detect TNF-α expression. Subsequently, negative control sections were incubated with PBS instead of primary antibody under the same environment and conditions (Gur and Timurkaan 2016b; Aktas and Gur 2022). Following the procedures described by the previous publications, sections were finally examined with an Olympus BX-53 (Tokyo, Japan) microscope and digital camera (DP 80, Olympus).

### Statistical analysis

IBM version 25.0 SPSS software package (SPSS Inc., Chicago, IL, USA) was utilized to analyse the quantitative data. Since the variable’s normal distribution was verified using the Shapiro–Wilk test, the data was statistically analysed by means of the parametric test. The one-way ANOVA and Tukey’s post hoc tests were then employed to determine the differences between groups. The mean ± standard deviation was used to express the data. Results were considered statistically significant at *p* < 0.05.

## Results

### Biochemical results

**Fig. 2:**
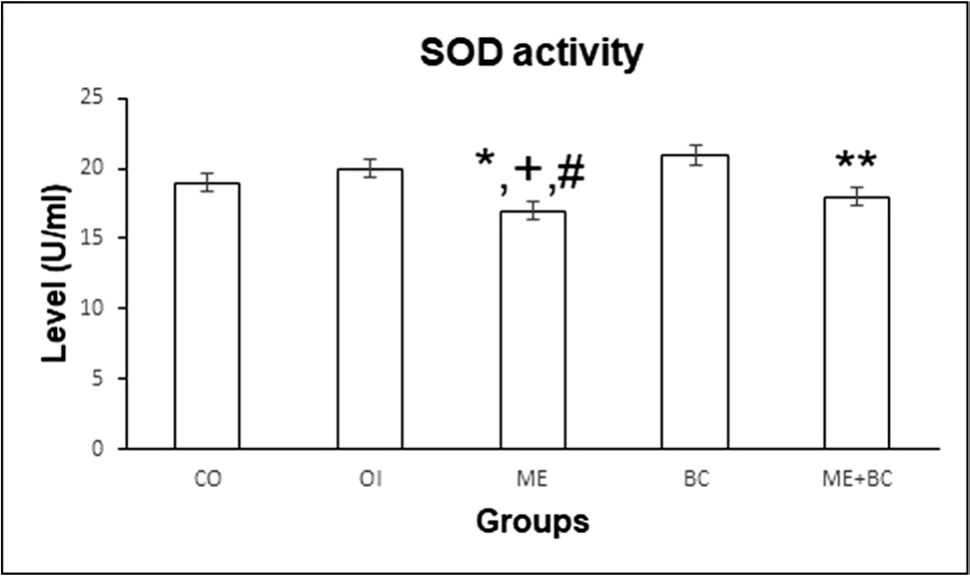
SOD levels in all groups. *, significantly different from the CO group; + , significantly different from the OI group; #, significantly different from the BC group; **, significantly different from ME. CO, control; OI, olive oil; ME, mercury chloride; BC, β-caryophyllene; ME + BC, mercury chloride + β-caryophyllene

SOD activities in all groups are shown in Fig. 2. The *F*-value between groups was 5.31. The results of the biochemical analysis revealed that the ME group’s SOD level was significantly lower than the CO (*p* < 0.05), OI (*p* < 0.05), and BC (*p* < 0.01) groups. Furthermore, the SOD level in the ME + BC group was significantly higher compared to the ME group (*p* < 0.05).

### Stereological results of the cerebellum

#### The number of granular cells

**Fig. 3:**
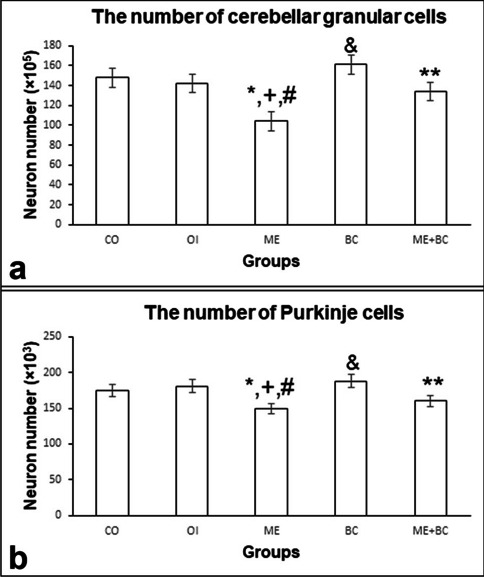
The number of cerebellar granular cells (**a**) and Purkinje cells (**b**) in all groups. *, significantly different from the CO group; + , significantly different from the OI group; #, significantly different from the BC group; &, significantly different from the ME + BC group; **, significantly different from ME. CO, control; OI, olive oil; ME, mercury chloride; BC, β-caryophyllene; ME + BC, mercury chloride + β-caryophyllene

The number of cerebellar granular cells in all groups is shown in Fig. 3a. The *F*-value between groups was 15.51. The number of granular cells in the ME group was detected to be significantly lower compared to the CO (*p* < 0.01), OI (*p* < 0.01), and BC (*p* < 0.01) groups. In the ME + BC group, the number of granular cells was also significantly higher compared to the ME group (*p* < 0.01). We also found a significant decrease in the ME + BC group in comparison with the BC group (*p* < 0.05).

#### The number of Purkinje cells

The number of Purkinje cells in all groups is shown in Fig. 3b. The *F*-value between groups was 8.87. Our findings revealed a statistically significant decrease in the number of Purkinje cells in the ME group compared to the CO (*p* < 0.01), OI (*p* < 0.01), and BC (*p* < 0.01) groups. When compared to the ME group, the number of Purkinje cells was significantly higher in ME + BC group (*p* < 0.05). A significant decrease was also detected in the ME + BC group compared to the BC group.

#### The mean volume of cerebellar grey matter

**Fig. 4:**
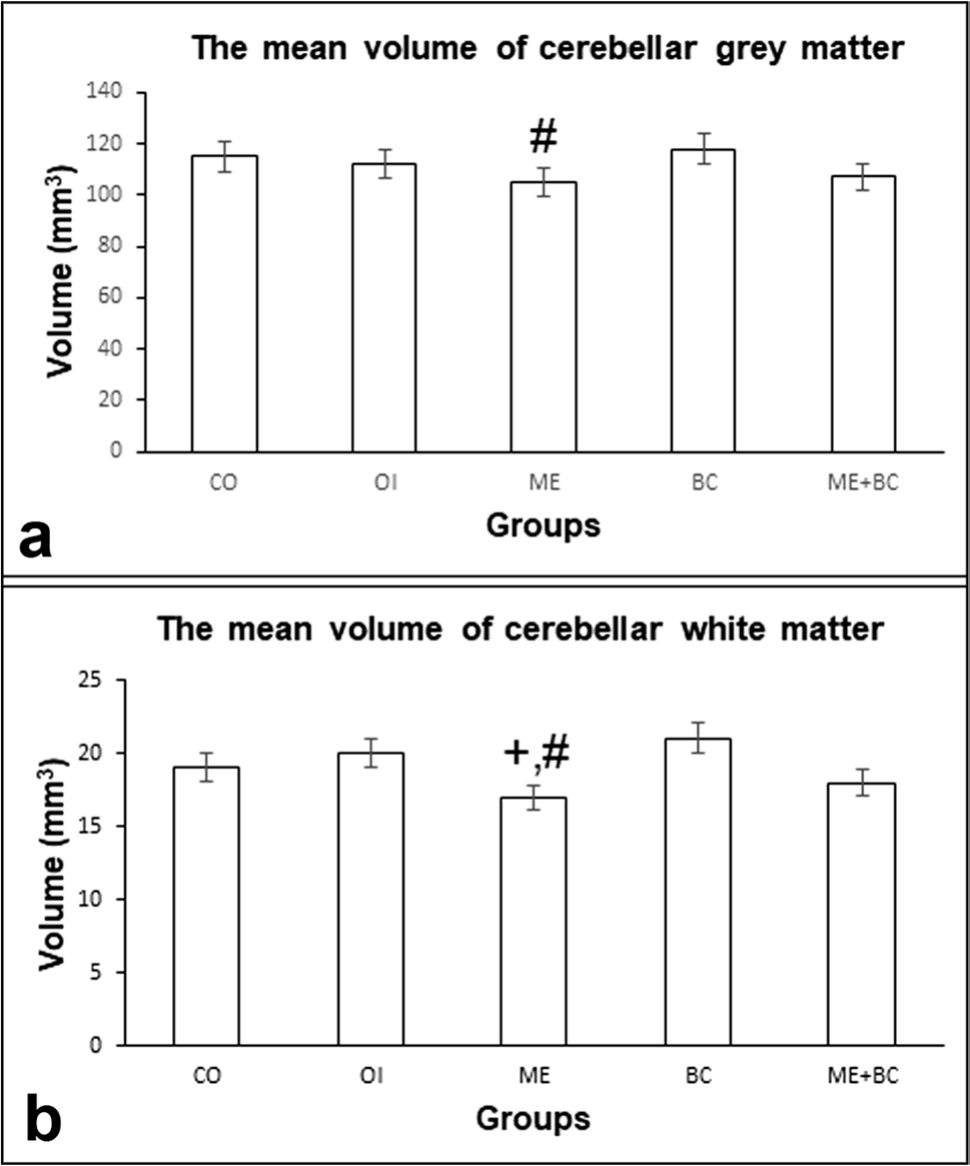
The mean volumes of cerebellar grey matter (**a**) and white matter (**b**) in all groups. + , significantly different from the OI group; #, significantly different from BC group. CO, control; OI, olive oil; ME, mercury chloride; BC, β-caryophyllene; ME + BC, mercury chloride + β-caryophyllene

The mean volumes of cerebellar grey matter in all groups are shown in Fig. 4a. The *F*-value between groups was 3.75. The mean grey matter volume was lower in the ME group compared to the CO group, but the difference was not statistically significant. In comparison to the BC group, there was also a significant decrease in the ME group (*p* < 0.05).

#### The mean volume of cerebellar white matter

The mean volumes of cerebellar white matter in all groups are shown in Fig. 4b. The *F*-value between groups was 5.77. Given our findings, the ME group’s mean volume of cerebellar white matter was significantly lower than that of the OI (*p* < 0.05) and BC (*p* < 0.01) groups.

#### The mean volume of the cerebellum

**Fig. 5:**
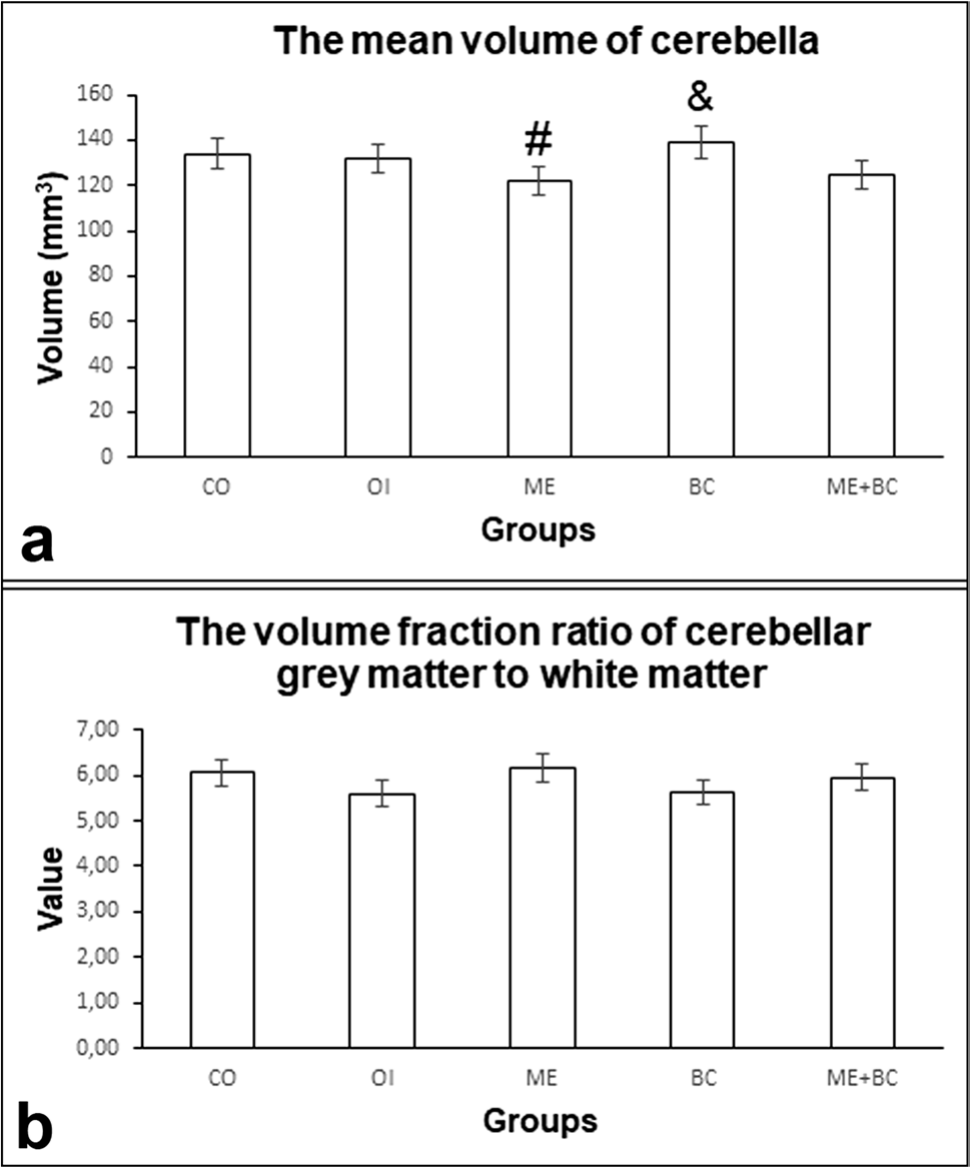
The mean volumes of cerebella (**a**) and the volume fraction ratios of cerebellar grey matter to white matter (**b**) in all groups. #, significantly different from the BC group; &, significantly different from the ME + BC group. CO, control; OI, olive oil; ME, mercury chloride; BC, β-caryophyllene; ME + BC, mercury chloride + β-caryophyllene

The mean volumes of the cerebella in all groups are shown in Fig. 5a. The *F*-value between groups was 4.94. Our study’s findings indicated that no significant difference was found between the ME and CO groups. Meanwhile, the mean cerebellar volumes of the ME and ME + BC groups were significantly lower than those of the BC group (*p* < 0.01).

#### The volume fraction ratio of cerebellar grey matter to white matter

The volume ratios of cerebellar grey matter to white matter are shown in Fig. 5b. The *F*-value between groups was 1.66. Statistical analysis of the data detected no significant difference in the volume ratio of cerebellar grey matter to white matter among all groups.

### Stereological results of spinal cord

#### The number of spinal motor neurons

**Fig. 6:**
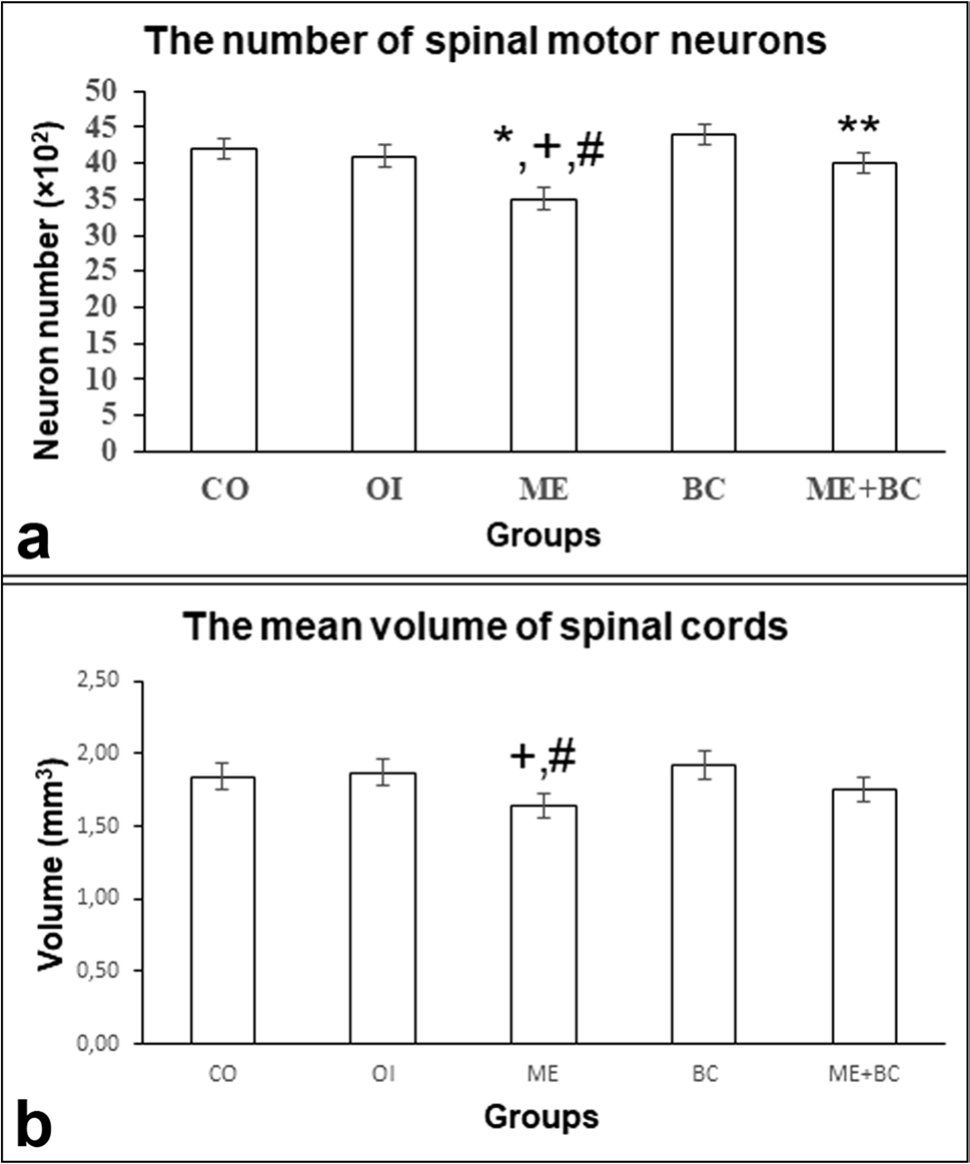
The number of spinal motor neurons (**a**) and the mean volumes of spinal cords (**b**) in all groups. *, significantly different from the CO group; + , significantly different from the OI group; #, significantly different from the BC group; **, significantly different from ME. CO, control; OI, olive oil; ME, mercury chloride; BC, β-caryophyllene; ME + BC, mercury chloride + β-caryophyllene

The number of spinal motor neurons in all groups is shown in Fig. 6a. The *F*-value between groups was 8.25. We detected a statistically significant reduction in the number of spinal motor neurons in the ME group compared to the CO (*p* < 0.01), OI (*p* < 0.01), and BC (*p* < 0.01) groups. The ME + BC group indicated a significantly higher number of motor neurons (*p* < 0.05) than the ME group (*p* < 0.05).

#### The mean volume of the spinal cord

The mean volumes of spinal cords in all groups are shown in Fig. 6b. The *F*-value between groups was 5.12. Our results revealed a significant reduction in the mean volume of spinal cord in the ME group compared to the OI (*p* < 0.05) and BC (*p* < 0.01) groups.

#### The mean volume of spinal grey matter

**Fig. 7:**
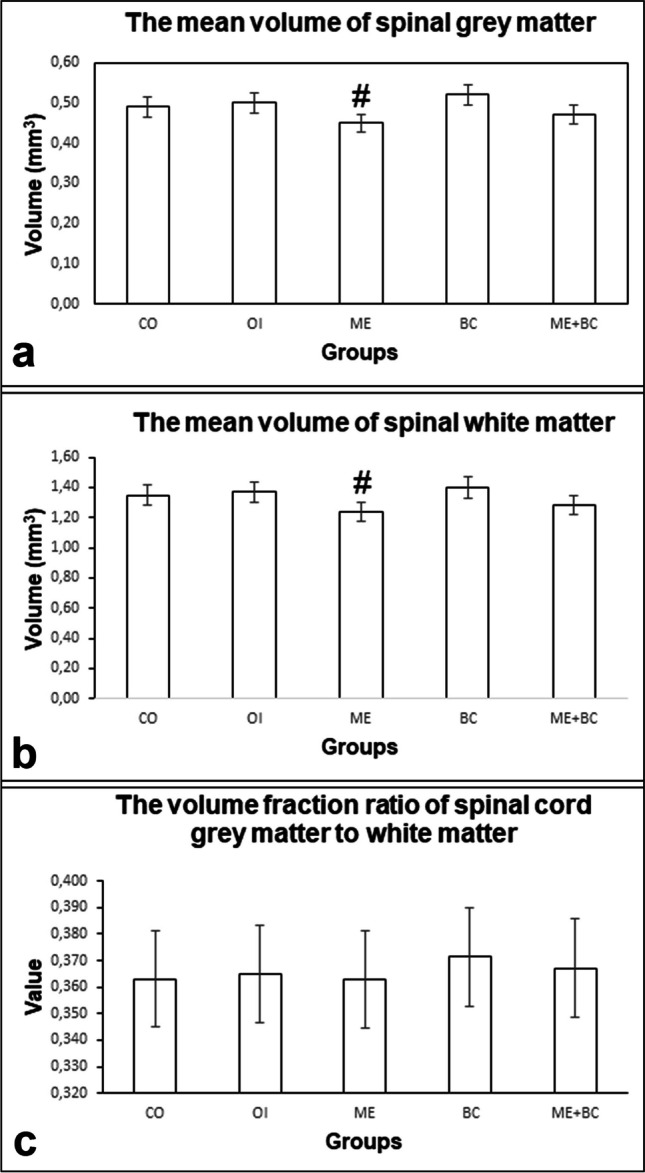
The mean volumes of spinal grey matter (**a**) and white matter (**b**), as well as the volume fraction ratios of spinal white matter to grey matter (**c**) in all groups. #, significantly different from the BC group. CO, control; OI, olive oil; ME, mercury chloride; BC, β-caryophyllene; ME + BC, mercury chloride + β-caryophyllene

The mean volumes of spinal grey matter in all groups are shown in Fig. 7a. The *F*-value between groups was 3.06. We determined a significant reduction in the mean volume of grey matter in the ME group than the BC group (*p* < 0.05). Besides, no significant difference was identified between the CO and ME groups.

#### The mean volume of spinal white matter

The mean volumes of spinal white matter in all groups are shown in Fig. 7b. The *F*-value between groups was 3.65. No significant difference in the mean volume of spinal white matter was found in the ME group than the CO group. We also detected a significant decrease in the ME group than the BC group (*p* < 0.05).

#### The volume fraction ratio of spinal grey matter to white matter

The volume fraction ratios of spinal white matter to grey matter in all groups are shown in Fig. 7c. The *F*-value between groups was 0.11. The findings of our study indicated that there was not a statistically significant difference among the groups.

### Histopathological results

**Fig. 8:**
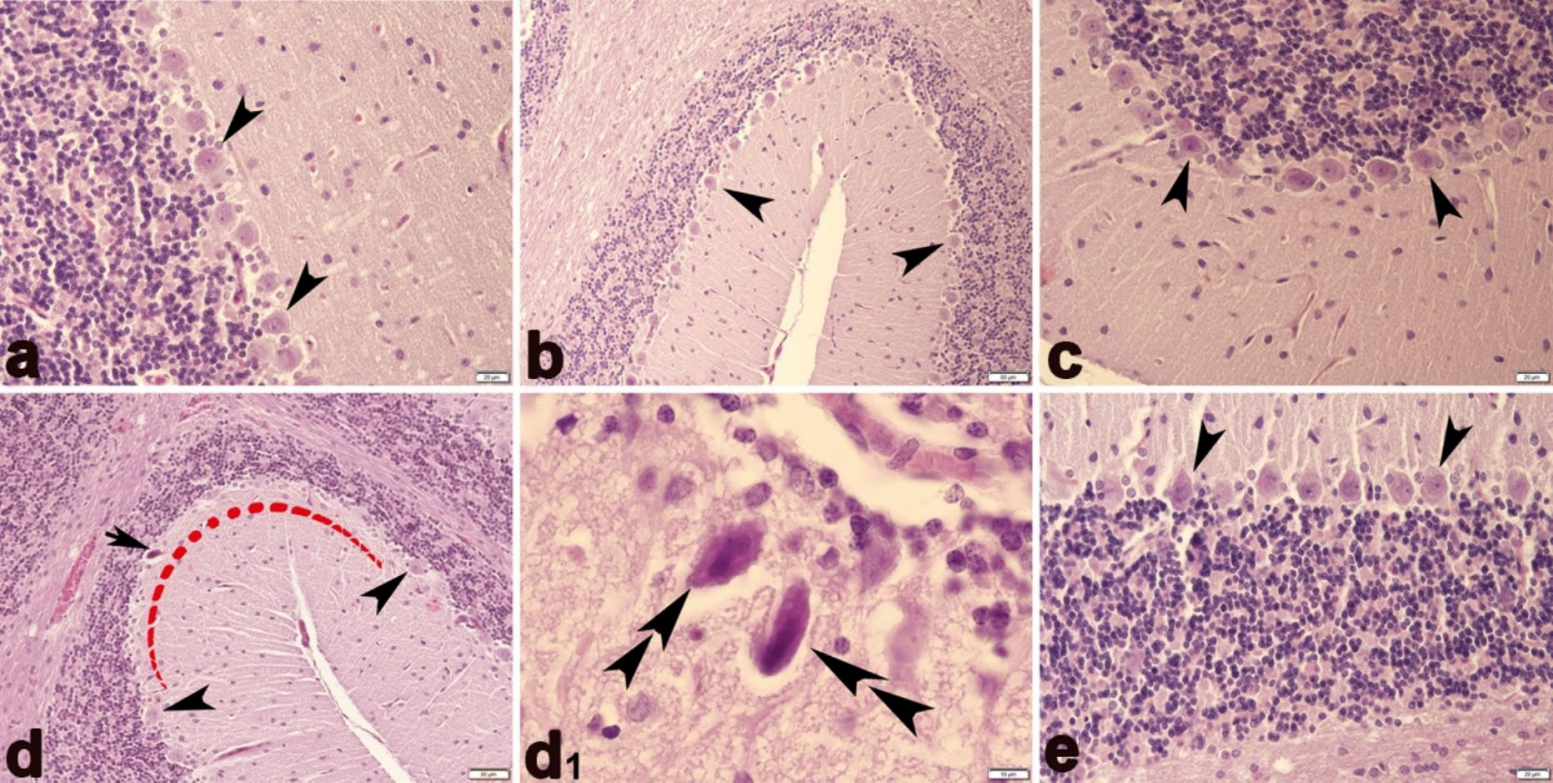
Haematoxylin and eosin staining of cerebellum sections of CO (**a**), OI (**b**), BC (**c**), ME (**d** and **d**_**1**_), and ME + BC (e) groups. Arrowhead, normal Purkinje cells with round nuclei; arrow, shrunken degenerate Purkinje cell with eosinophilic cytoplasm and condensed nucleus; double arrowhead, Shrinkage in Purkinje cells, eosinophilia in the cytoplasm, condensation, bending, and elongation in the nucleus; red arch-shaped region, Purkinje cell loss (**a** and **c** × 400; **b**, **d**, and **e** × 200; **d**_**1**_ × 1000). CO, control; OI, olive oil; ME, mercury chloride; BC, β-caryophyllene; ME + BC, mercury chloride + β-caryophyllene

The cerebellar tissue of the CO, OI, and BC groups exhibited normal architectures of white matter, molecular layer, Purkinje, and granule cell layers (Fig. 8a–c). In contrast, the cerebellar tissues in the ME group revealed evidence of the absence and degeneration of Purkinje cells (Fig. 8d and d1). These degenerated cells showed morphological alterations, including shrinkage, cytoplasmic eosinophilia, and nuclear condensation, elongation, and bending. In the ME + BC group, histopathological alteration was ameliorated in the cerebellar tissue (Fig. 8e).

**Fig. 9:**
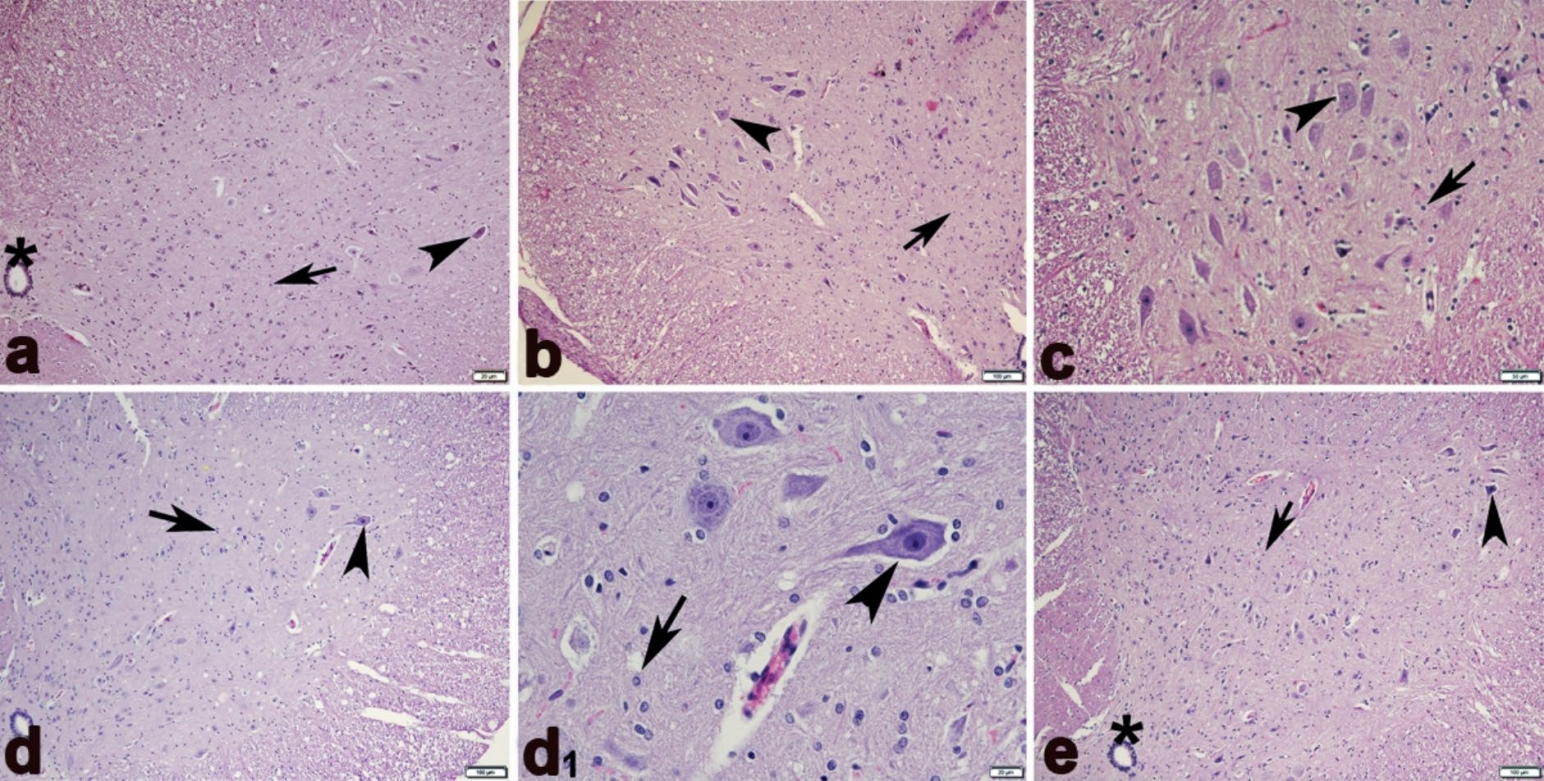
Haematoxylin and eosin staining of spinal cord sections of CO (**a**), OI (**b**), BC (**c**), ME (**d** and **d**_**1**_), and ME + BC (**e**) groups. Arrowhead, motor neurons; arrow, neuroglial cells; asterisks, ependymal cells (**a**, **b**, **d**, and **e** × 100; **c** × 200; **d**_**1**_ × 400). CO, control; OI, olive oil; ME, mercury chloride; BC, β-caryophyllene; ME + BC, mercury chloride + β-caryophyllene

We also detected that the histological structures of the spinal cord tissue were also similar in BC, OI, and CO groups (Fig. 9a–c). Compared to the CO group, the ME group’s spinal anterior horn exhibited a slight decline in motor neuron density (Fig. 8d and d1); however, alteration in the ME + BC group was improved (Fig. 9e).

### Immunohistochemical results

**Fig. 10:**
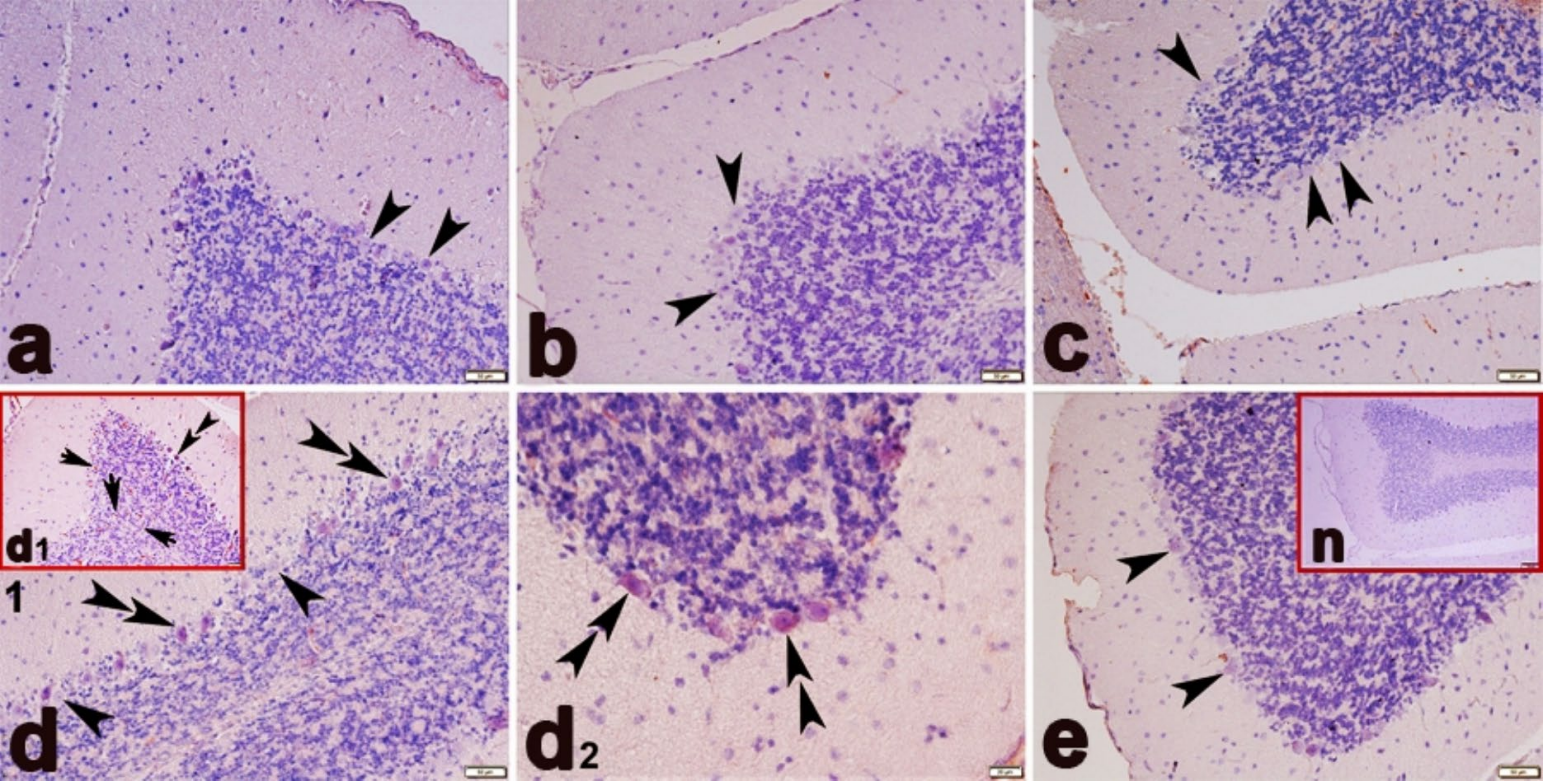
Immunohistochemical expression of TNF-α in the cerebellum of the CO (**a**), OI (**b**), BC (**c**), ME (**d**, d_1_, and **d**_**2**_), and ME + BC (**e**). groups. Double arrowhead, TNF-α positive Purkinje cells; arrowhead, TNF-α negative Purkinje cell; arrow, TNF-α positive neuroglial cells; N, negative control (**a**, **b**, **c**, **d**, **d**_1_, and **e** × 200; **d**_**2**_ × 400). CO, control; OI, olive oil; ME, mercury chloride; BC, β-caryophyllene; ME + BC, mercury chloride + β-caryophyllene

Analysis of TNF-α immunostaining detected that the cerebellar tissues of the CO (a), OI (b), and BC (c) groups were TNF-α-negative. On the contrary, TNF-α-positive immunostaining was detected in the ME group (Fig. 10d, d1, and d2). In this group, TNF-α positive was evident in Purkinje and neuroglial cells; however, the most prominent immunostaining was identified in the cytoplasm of Purkinje cells. TNF-α immunostaining was also improved in the cerebellar tissues of the ME + BC group compared to the ME group (Fig. 10e).

**Fig. 11:**
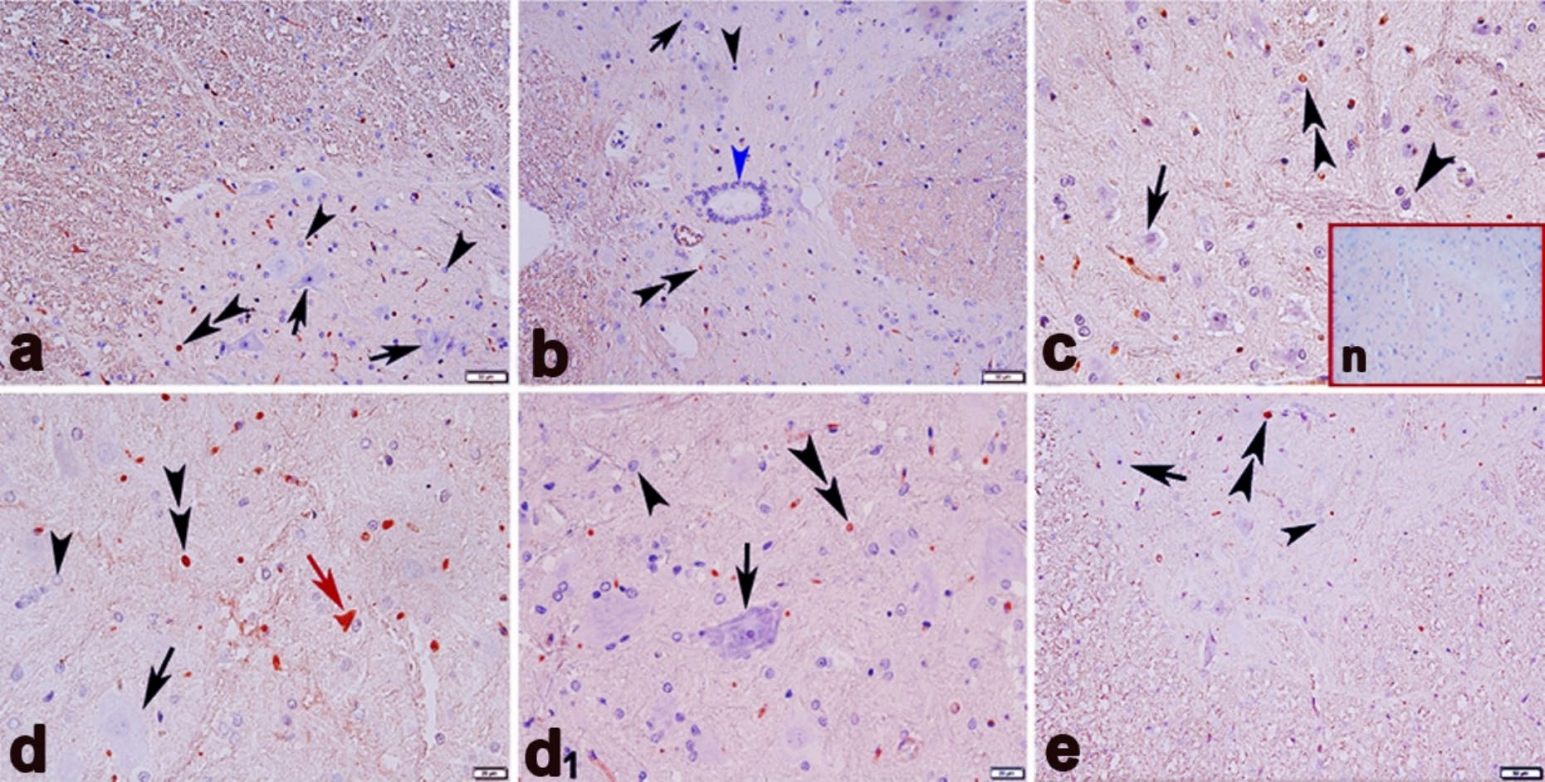
Immunohistochemical expression of TNF-α in the spinal cord of the CO (**a**), OI (**b**), BC (**c**), ME (**d** and **d**_**1**_), and ME + BC (**e**) groups. Double arrowhead, TNF-α positive neuroglial cells; arrowhead, TNF-α negative neuroglial cells; black arrow, TNF-α negative ventral horn neuron; red arrow, TNF-α positive ventral horn neuron; blue arrowhead, TNF-α negative ependymal cells; n, negative control (**a**, **b**, and **e** × 200; **c**, **d**, and **d**_**1**_ × 400). CO, control; OI, olive oil; ME, mercury chloride; BC, β-caryophyllene; ME + BC, mercury chloride + β-caryophyllene

In the spinal cord tissues, slight TNF-α-positive immunostaining of neuroglial cells was detected in the CO (a), OI (b), and BC (c) groups. In the ME group, more TNF-α positive neuroglial cells were found, whereas they were less in the CO group (Fig. 11d and d1). In the ME group, TNF-α-positive was also observed in the motor neurons in the anterior horn. Besides, there was a discernible improvement of immunostaining in the ME + BC group (Fig. 11e).

## Discussion

Mercury compounds are highly hazardous, a characteristic that makes it unique among heavy metals. Environmental exposure to mercury can occur through heavy metal smelting, chemical production, industrial mining, significant agricultural use, and solid waste disposal (Tchounwou et al. 2012). Hence, long-term exposure to ME has raised public health concerns worldwide. Extensive studies have recently been carried out on the issue of protecting living organisms against environmental pollutants.

Our biochemical analysis revealed that following exposure to ME, the SOD level in the ME group was significantly elevated than in the CO and OI groups. This finding aligned with earlier research that documented the detrimental impact of ME (2 mg/kg) on the biological functioning of antioxidant enzymes in adult rats (Said et al. 2021). The endogenous antioxidant enzymes contribute significantly to the preservation of oxidative balance through the elimination of free radicals from the organism’s body. A disruption in the oxidative balance may give rise to an excess of ROS (Yahyazadeh et al. 2018; Wang et al. 2021). Consequently, these essential macromolecules suffer oxidative damage due to the antioxidant enzymes’ inability to scavenge ROS, extremely unstable molecules. Cellular architecture and function can also be compromised by such biomolecules that have undergone oxidative damage (Nawrot et al. 2011). Earlier research reported oxidative damage to the cerebral and cerebellar tissues of rats due to decreased SOD and GSH levels owing to exposure to ME at a dose of 4 mg/kg (Owoeye et al. 2019). In the ME + BC group, decreased SOD activity also revealed the potential of BC as an antioxidant. The main reason for BC’s neuroprotective efficacy may be its ability to mitigate ME-induced oxidative stress (Kanojia et al. 2021). A previous investigation on a rat model showed that administration of 50 mg/kg BC strengthened the antioxidant defence system such as SOD, catalase (CAT), and glutathione (Ojha et al. 2016). Furthermore, there was a relationship between BC treatment and diminished levels of lipid peroxidation, malondialdehyde (MDA), inducible nitric oxide synthase, and cyclooxygenase-2, which in turn improved neurodegeneration. Mitochondria can generate the majority of intracellular ROS, which induces dysfunction through damage to its essential components. Moreover, the vulnerability of neurons to mitochondria-induced oxidative injury is further increased by diminished enzymatic antioxidant status (Kumar 2016). BC’s neuroprotective benefits at doses of 102 and 306 mg/kg have also been associated with the amelioration of mitochondrial dysfunction via restoring the activity of enzymatic antioxidants like SOD and CAT, as well as alleviating lipid peroxidation and MDA (Lou et al. 2016; Ullah et al. 2021).

The study’s stereological findings also indicated that administration of ME significantly reduced the number of spinal cord motor neurons, cerebellar granular cells, and Purkinje cells in the ME group compared to the CO and OI groups. The neurotoxicity of ME could be the manifestation of its potential risk on the spinal cords and cerebellar tissues. Substantial evidence has shown an association between exposure to environmental contaminants and impairment in a vital component of the CNS. ME-induced oxidative stress may be the main cause of damage to the CNS. Oxidative damage to healthy neurons has been proposed as the consequence of lipid peroxidation and mitochondrial dysfunction arising from excessive ROS generation (Slimen et al. 2014; Behzadfar et al. 2020). Meanwhile, the CNS neurons are also at greater risk of damage from oxidation due to their high oxygen consumption and lipid-rich composition (Salim 2017). The interaction between DNA and ROS has the capability to induce cell damage or death by disrupting DNA structures such as protein crosslinks, base lesions, and single- and double-strand breaks (Fujii and Tsunoda 2011; Dizdaroglu and Jaruga 2012). A research study on adult rats reported a decline in the number of both motor and sensory neurons in the tissues of the cervical spinal cord after exposure to 0.375 mg/kg ME, which is in line with our results (Correa et al. 2020). There have also been reports of neuronal deaths in the tissues of the spinal cord and cerebellum following ME administration (Correa et al. 2020; Otong et al. 2022). As a result of unsaturated fatty acid oxidation, lipid peroxidation was shown to be markedly elevated in the CNS of rats treated with 2 and 4 mg/kg ME (Rao et al. 2010). Increased lipid peroxidation has been documented to be associated with neurodegeneration (Angelova et al. 2021). The reaction of ME with carboxyl and phosphoryl groups also causes a malfunction of main structures and biomolecules such as membranes, structural proteins, and enzymes (Cappelletti et al. 2019). Moreover, synaptic transmission disruption triggered by 20 and 100 micromole ME exposure has been reported to impair the physiological neuronal activities in rats, which may be derived from the complete blockade of acetylcholine (Cooper and Manalis 1983; Yuan and Atchison 1994). Another possible cause of ME’s neurotoxicity is the disruption of the calcium adenosine triphosphate pump, followed by an imbalance in calcium homeostasis (Gasso et al. 2001; Paduraru et al. 2022).

ME can be absorbed following skin and intravenous, as well as the oral route of exposure which is the most prevalent. The rate of absorption through ingestion has been estimated to be approximately 7–15% in mice, which is dependent on various factors including pH in the intestines, dietary type, and age (Walsh 1982; Endo et al. 1990; Wu et al. 2024). After being absorbed, inorganic mercury can be distributed throughout the body and accumulated in a variety of vital organs (Cappelletti et al. 2019). ME’s poor lipid solubility makes it more difficult to cross the blood–brain barrier. Although there is a smaller amount of ME in the CNS, it has the longest retention time. Metabolism of ME occurs in the body through oxidation and reduction. After exposure, ME is oxidized to a divalent cation, which is subsequently reduced to a metallic or monovalent form by glutathione reductase and exhaled as mercury vapor (Cappelletti et al. 2019). However, main elimination of ME is through the urine and faeces (Park and Zheng 2012).

The CNS’s ionic microenvironment is also preserved by the blood–brain barrier, which serves as a selectively semipermeable border. Disruption of this specialized system may contribute to the unproper functioning of neurons following disturbance in CNS haemostasis (Abu-Zeid et al. 2021). The BBB’s integrity is thought to be compromised by administration of 6.7 mg/kg ME due to oxidative damage, inflammation, and reduced mRNA expression of the tight junction protein genes in rats (Abu-Zeid et al. 2021). The conversion of ME to methylmercury is another reason for easy penetration of mercury into the blood–brain barrier and accumulation in the CNS (Xu et al. 2012). Therefore, neuronal damage may occur due to the entry of ME into the CNS and subsequent absorption by neurons (Pamphlett and Waley 1996). Even at 5–250 micromole concentrations, ME has been found to inhibit membrane adenosine deaminase activity (Senger et al. 2010). Dysfunction of the brain and cerebellum has also been attributed to the depletion of enzymatic activity triggered by exposure to ME (Rao et al. 2010).

We also found that treatment of BC eventually led to a significantly higher number of Purkinje cells, granular cells, and motor neurons in the ME + BC group compared to the ME group. These results revealed the antioxidant potential and neuroprotective effect of BC on the spinal cord and cerebellar tissues exposed to ME. Since environmental exposure to ME is considered a threat to healthy life, researchers are interested in finding ways to mitigate the problem caused by these harmful compounds. Dietary antioxidant supplements have been the focus of numerous scientific research as a critical strategy for improving ME-induced problems (Ullah et al. 2021). Vital cells and organs have been proven to be protected from oxidative damage by BC, a safe natural antioxidant (Al-Taee et al. 2019). Given its neuroprotective capacities, BC may be employed as a food ingredient to help improve the architecture and function of the CNS (Sudeep et al. 2021). BC administration has also been reported to inhibit the intrinsic pathway of apoptosis by upregulation of Bcl-2 gene expression (Yovas et al. 2022). In the CNS, treatment with BC can boost antioxidant enzyme activity and reduce lipid peroxidation (Kanojia et al. 2021).

The present histopathological examination of the ME group revealed abnormalities such as loss and degeneration of Purkinje cells. Degenerated Purkinje cells also exhibited prominent alterations, including elongation, bending, nuclear condensation, and cytoplasmic eosinophilia. The spinal cord’s motor neuron density was also detected to be slightly reduced. These findings confirmed the toxic potential of ME on the cerebellar and spinal cord tissues, which was consistent with the results of the previous studies (Altunkaynak et al. 2019; Owoeye et al. 2019; Correa et al. 2020; Otong et al. 2022; Shalan 2022). A possible mechanism of ME-induced neurodegeneration may be through selective suppression of neuronal cytokine-mediated Janus kinase following the development of pro-oxidant activity (Monroe and Halvorsen 2009). A study also documented the potential toxicity of ME at a dose of 10 − 5 mol on membrane integrity of neurons and glia cells in culture (Walum and Marchner 1983). Another potential cause of mercury toxicity is a reduction in nerve growth factor, which impacts the development of neurons in the CNS (Soderstrom and Ebendal 1995). Oxidative stress induced by ME can also trigger damage to DNA, respiratory chain, membrane permeability, and defence systems in mitochondria, followed by neurodegeneration (Guo et al. 2013; Said et al. 2021). In the ME + BC group, BC was possibly responsible for the improvement of the cerebellar and spinal cord alteration after ME exposure. The potential capability of BC may be attributed to the improvement of enzymatic antioxidant and consequently oxidative state (Ames-Sibin et al. 2018). Microglia and astrocytes are known to be involved in neuroinflammation and oxidative stress in the CNS. According to the findings of an experimental trial, BC administration at a dose of 50 mg/kg significantly reduced the activity of astrocytes and microglia exposed to oxidative stress and inflammation (Ojha et al. 2016). Accordingly, this situation prompted the preservation of neurons and improvement of neurodegeneration.

Given the present immunohistochemical analysis, ME exposure upregulated TNF-α expression, which in turn triggered an inflammatory response within the spinal cord and cerebellum tissues of the ME group. This finding revealed a relationship between ME exposure and high TNF-α expression, which is another indication of neuroinflammation caused by oxidative stress. In response to a biological damage, TNF-α, a proinflammatory cytokine, is thought of as an essential factor involved in abnormality in the cerebellar and spinal cord tissues. In addition to being produced by a variety of cells in the body, TNF-α in the CNS is synthesized by microglia, astrocytes, and some neurons (da Costa et al. 2020). The majority of proinflammatory and apoptotic responses are triggered by TNF-R1 receptor, followed by the activation of numerous intracellular pathways. Binding of TNF-α to TNF-R1 receptor can modulate inflammation, proliferation, and apoptosis (MacEwan 2002; Sprowl et al. 2012). This interaction stimulates both signalling pathways including mitogen-activated protein kinase and nuclear factor-κB, which results in inflammation (van Loo and Bertrand 2023). TNF-α also causes astrocytes and neurons to undergo apoptosis by activating the caspase-3 (Zhao et al. 2001). This neurotoxic and inflammatory mediator, TNF-α, has been reported to promote neurodegeneration and neuroinflammation after releasing from glial cells and neurons. TNF-a in the periphery can also cross the impaired blood–brain barrier, then cause complication in the CNS (Kempuraj et al. 2016). Considering the previous research on the mouse CNS, oxidative stress following ME (4 mg/kg)-induced reduction in enzymatic antioxidant capacity has been reported to be the cause of the upregulation of TNF-α expression (Gu et al. 2023). In this condition, the excess of neuroinflammation occurs and may be accompanied by the potential risk of neurodegeneration. Furthermore, increased permeability of the blood–brain barrier has been associated with higher expression level TNF-α (Mayhan 2002). In the ME + BC group, BC supplementation attenuated inflammation status in the spinal cord and cerebellum tissues owing to downregulation of TNF-α, which suggests its putative anti-inflammatory capability. Earlier experimental research documented that BC activated cannabinoid 2 receptor expressed in the various regions of the CNS and preserved nervous tissues (Chen et al. 2017; Irrera et al. 2020). Consequently, TNF-α expression is suppressed by activating peroxisome proliferator–activated receptor gamma and decreasing NF-κB (Irrera et al. 2020).

To the best of our knowledge, this study was carried out for the first time to survey whether BC administration could minimize the potential health risk of ME on the spinal cord and cerebellar tissues. Further research with additional parameters should be conducted for gathering more information, as each parameter possesses a significant impact on quantitative studies. We also suggested investigating alternative dietary supplements which exhibit broad-spectrum antioxidant capabilities.

## Conclusion

Our quantitative findings revealed that the detrimental impact of ME exposure was associated with a significant increase in SOD level, as well as a significant decrease in the number of Purkinje cells, granular cells, and spinal motor neurons. Elevated TNF-α-positive immunostaining also indicated an inflammatory response to ME treatment. The results of the stereological, immunohistochemical, histological, and biochemical analyses were compatible with each other and revealed that exposure to ME caused damage to the cerebellar and spinal cord tissues. Administration of BC, a potential antioxidant, helped mitigate the ME-induced alterations in the cerebellum and spinal cords. For this reason, BC might be an efficient therapeutic alternative as a medicine or supplement in the clinical treatment of neurological complication induced by ME.

## Data Availability

The datasets used and/or analysed during the current study are available from the corresponding author on reasonable request.
